# Improved upper limb function in non-ambulant children with SMA type 2 and 3 during nusinersen treatment: a prospective 3-years SMArtCARE registry study

**DOI:** 10.1186/s13023-022-02547-8

**Published:** 2022-10-23

**Authors:** Astrid Pechmann, Max Behrens, Katharina Dörnbrack, Adrian Tassoni, Franziska Wenzel, Sabine Stein, Sibylle Vogt, Daniela Zöller, Günther Bernert, Tim Hagenacker, Ulrike Schara-Schmidt, Maggie C. Walter, Astrid Bertsche, Katharina Vill, Matthias Baumann, Manuela Baumgartner, Isabell Cordts, Astrid Eisenkölbl, Marina Flotats-Bastardas, Johannes Friese, René Günther, Andreas Hahn, Veronka Horber, Ralf A. Husain, Sabine Illsinger, Jörg Jahnel, Jessika Johannsen, Cornelia Köhler, Heike Kölbel, Monika Müller, Arpad von Moers, Annette Schwerin-Nagel, Christof Reihle, Kurt Schlachter, Gudrun Schreiber, Oliver Schwartz, Martin Smitka, Elisabeth Steiner, Regina Trollmann, Markus Weiler, Claudia Weiß, Gert Wiegand, Ekkehard Wilichowski, Andreas Ziegler, Hanns Lochmüller, Janbernd Kirschner, SMArtCARE study group, Lisa Ameshofer, Barbara Andres, Daniela Angelova-Toshkina, Daniela Banholzer, Christina Bant, Petra Baum, Sandra Baumann, Ute Baur, Benedikt Becker, Bettina Behring, Julia Bellut, Andrea Bevot, Jasmin Bischofberger, Lisa Bitzan, Bogdan Bjelica, Markus Blankenburg, Sandra Böger, Friederike Bonetti, Anke Bongartz, Svenja Brakemeier, Lisa Bratka, Nathalie Braun, Sarah Braun, Brigitte Brauner, Christa Bretschneider, Nadine Burgenmeister, Bea Burke, Sebahattin Cirak, Andrea Dall, Heike de Vries, Adela Della Marina, Jonas Denecke, Marcus Deschauer, Zylfie Dibrani, Uta Diebold, Lutz Dondit, Jessica Drebes, Joenna Driemeyer, Vladimir Dukic, Matthias Eckenweiler, Mirjam Eminger, Michal Fischer, Cornelia Fischer, Maren Freigang, Philippa Gaiser, Andrea Gangfuß, Stephanie Geitmann, Annette George, Magdalena Gosk-Tomek, Susanne Grinzinger, Kristina Gröning, Martin Groß, Anne-Katrin Güttsches, Anna Hagenmeyer, Hans Hartmann, Julia Haverkamp, Miriam Hiebeler, Annegret Hoevel, Georg Friedrich Hoffmann, Britta Holtkamp, Dorothea Holzwarth, Annette Homma, Viola Horneff, Carolin Hörnig, Anna Hotter, Andrea Hubert, Peter Huppke, Eva Jansen, Lisa Jung, Nadja Kaiser, Stefan Kappel, Bolte Katharina, Johannes Koch, Stefan Kölke, Brigitte Korschinsky, Franziska Kostede, Karsten Krause, Hanna Küpper, Annina Lang, Irene Lange, Thorsten Langer, Yvonne Lechner, Helmar Lehmann, Christine Leypold, Paul Lingor, Jaqueline Lipka, Wolfgang Löscher, Antje Luiking, Gerrit Machetanz, Eva Malm, Kyriakos Martakis, Bettina Menzen, Moritz Metelmann, Gerd Meyer zu Hörste, Federica Montagnese, Kathrin Mörtlbauer, Petra Müller, Anne Müller, Anja Müller, Lars Müschen, Christoph Neuwirth, Moritz Niesert, Josefine Pauschek, Elke Pernegger, Susanne Petri, Veronika Pilshofer, Barbara Plecko, Jürgen Pollok, Martin Preisel, Manuel Pühringer, Anna Lisa Quinten, Sabine Raffler, Barbara Ramadan, Mika Rappold, Christian Rauscher, Kerstin Reckmann, Tabea Reinhardt, Melanie Röder, Doris Roland-Schäfer, Erdmute Roth, Lena Ruß, Afshin Saffari, Mareike Schimmel, Melina Schlag, Beate Schlotter-Weigel, Joanna Schneider, Jan-Christoph Schöne-Bake, David Schorling, Isabella Schreiner, Stephanie Schüssler, Michaela Schwarzbach, Michaela Schwippert, Luisa Semmler, Karin Smuda, Alina Sprenger-Svacina, Theresa Stadler, Paula Steffens, Daniela Steuernagel, Benjamin Stolte, Corinna Stoltenburg, Gehrke Tasch, Andreas Thimm, Elke Tiefenthaler, Raffi Topakian, Matthias Türk, Lieske van der Stam, Katia Vettori, Peter Vollmann, Matthias Vorgerd, Deike Weiss, Stephan Wenninger, Svea Werring, Maria Wessel, Ute Weyen, Sabine Wider, Nils Ole Wiebe, Anna Wiesenhofer, Sarah Wiethoff, Corinna Wirner, Camilla Wohnrade, Gilbert Wunderlich, Daniel Zeller, Michael Zemlin, Joachim Zobel

**Affiliations:** 1 Department of Neuropediatrics and Muscle Disorders, Faculty of Medicine, Medical Center – University of Freiburg, University of Freiburg, Mathildenstr. 1, Freiburg, Germany; 2 Faculty of Medicine, Institute of Medical Biometry and Statistics, Medical Center – University of Freiburg, Freiburg, Germany; 3 Clinical Trials Unit, Faculty of Medicine, Medical Center – University of Freiburg, University of Freiburg, Freiburg, Germany; 4 Department of Pediatrics, Clinic Favoriten, Vienna, Austria; 5 Department of Neurology, Center for Translational Neuro‐ and Behavioral Sciences (C‐TNBS), University Medicine Essen, Essen, Germany; 6 Department of Neuropediatrics and Neuromuscular Centre for Children and Adolescents, Center for Translational Neuro- and Behavioral Sciences, University of Duisburg-Essen, Essen, Germany; 7 Department of Neurology, Friedrich-Baur-Institute, Ludwig-Maximilians-University of Munich, Munich, Germany; 8 University Hospital for Children and Adolescents, Ernst-Heydemann-Straße 8, 18057 Rostock, Germany; 9 Department of Pediatric Neurology and Developmental Medicine, LMU Center for Children With Medical Complexity, Dr. von Hauner Children’s Hospital, LMU Hospital, Ludwig-Maximilians-University, Munich, Germany; 10 Division of Pediatric Neurology, Department of Pediatrics I, Medical University of Innsbruck, Innsbruck, Austria; 11 Department of Pediatrics and Adulescent Medicine, Ordensklinikum Linz, Barmherzige Schwestern, Linz, Austria; 12 Department of Neurology, Technical University of Munich, School of Medicine, Munich, Germany; 13 Department of Paediatrics and Adolescent Medicine, Johannes Kepler University Linz, Kepler University Hospital, Krankenhausstrasse 26-30, 4020 Linz, Austria; 14 Department of Pediatric Neurology, Saarland University Hospital, Homburg, Germany; 15 Department of Neuropediatrics, University Hospital Bonn, Bonn, Germany; 16 Department of Neurology, University Hospital Carl Gustav Carus, Dresden, Germany; 17 Department of Child Neurology, Justus-Liebig University, Giessen, Germany; 18 Department of Paediatric Neurology, University Children’s Hospital, Tübingen, Germany; 19 Department of Neuropediatrics, Jena University Hospital, Jena, Germany; 20 Clinic for Pediatric Kidney-, Liver- and Metabolic Diseases, Hannover Medical School, Hannover, Germany; 21 Division of General Pediatrics, Department of Pediatrics and Adolescent Medicine, LKH Klagenfurt, Medical University of Graz, Graz, Austria; 22 Department of Pediatrics, University Medical Center Hamburg-Eppendorf, Hamburg, Germany; 23 Abteilung Für Neuropädiatrie Und Sozialpädiatrie, Universitätsklinik Für Kinder- Und Jugendmedizin, St. Josef-Hospital, Ruhr-Universität Bochum, Bochum, Germany; 24 Department of Neuropediatrics, University Children’s Hospital Würzburg, Würzburg, Germany; 25 Department of Pediatrics Und Neuropediatrics, DRK Kliniken Berlin, Berlin, Germany; 26 Division of General Pediatrics, Department of Pediatrics and Adolescent Medicine, Medical University of Graz, Graz, Austria; 27 Department for Pediatric Neurology, Center for Child and Adolescent Medicine Olgahospital, Psychosomatic and Pain Medicine, Child Pain Center Baden-Württemberg, Klinikum Stuttgart, Stuttgart, Germany; 28 Department of Pediatrics, State Hospital of Bregenz, Bregenz, Austria; 29 Department of Pediatric Neurology, Klinikum Kassel, Kassel, Germany; 30 Department of Pediatric Neurology, Münster University Hospital, Münster, Germany; 31 Abteilung Neuropaediatrie, Medizinische Fakultät Carl Gustav Carus, Technische Universität Dresden, Dresden, Germany; 32 Department of Pediatrics and Adolescent Medicine, Johannes Kepler University/Hospital, Linz, Austria; 33 Division of Pediatric Neurology, Department of Pediatrics, Friedrich-Alexander-University of Erlangen-Nürnberg, Erlangen, Germany; 34 Department of Neurology, Heidelberg University Hospital, Heidelberg, Germany; 35 Department of Pediatric Neurology and Center for Chronically Sick Children, Charité – University Medicine Berlin, Augustenburger Platz 1, Berlin, Germany; 36 Neuropediatrics Section of the Department of Pediatrics, Asklepios Clinic Hamburg Nord-Heidberg, Hamburg, Germany; 37 Department of Paediatrics and Pediatric Neurology, University Medical Centre, Georg August University Göttingen, Robert-Koch-Straße 40, 37075 Göttingen, Germany; 38 Department of Neuropediatrics and Metabolic Medicine, University Hospital Heidelberg, Heidelberg, Germany; 39 Children’s Hospital of Eastern Ontario Research Institute, Division of Neurology, Department of Medicine, The Ottawa Hospital; and Brain and Mind Research Institute, University of Ottawa, Ottawa, Canada

**Keywords:** Spinal muscular atrophy, Nusinersen, Sitter, Later-onset, SMArtCARE

## Abstract

**Background:**

The development and approval of disease modifying treatments have dramatically changed disease progression in patients with spinal muscular atrophy (SMA). Nusinersen was approved in Europe in 2017 for the treatment of SMA patients irrespective of age and disease severity. Most data on therapeutic efficacy are available for the infantile-onset SMA. For patients with SMA type 2 and type 3, there is still a lack of sufficient evidence and long-term experience for nusinersen treatment. Here, we report data from the SMArtCARE registry of non-ambulant children with SMA type 2 and typen 3 under nusinersen treatment with a follow-up period of up to 38 months.

**Methods:**

SMArtCARE is a disease-specific registry with data on patients with SMA irrespective of age, treatment regime or disease severity. Data are collected during routine patient visits as real-world outcome data. This analysis included all non-ambulant patients with SMA type 2 or 3 below 18 years of age before initiation of treatment. Primary outcomes were changes in motor function evaluated with the Hammersmith Functional Motor Scale Expanded (HFMSE) and the Revised Upper Limb Module (RULM).

**Results:**

Data from 256 non-ambulant, pediatric patients with SMA were included in the data analysis. Improvements in motor function were more prominent in upper limb: 32.4% of patients experienced clinically meaningful improvements in RULM and 24.6% in HFMSE. 8.6% of patients gained a new motor milestone, whereas no motor milestones were lost. Only 4.3% of patients showed a clinically meaningful worsening in HFMSE and 1.2% in RULM score.

**Conclusion:**

Our results demonstrate clinically meaningful improvements or stabilization of disease progression in non-ambulant, pediatric patients with SMA under nusinersen treatment. Changes were most evident in upper limb function and were observed continuously over the follow-up period. Our data confirm clinical trial data, while providing longer follow-up, an increased number of treated patients, and a wider range of age and disease severity.

## Background

Treatment and care of patients with spinal muscular atrophy (SMA) have changed dramatically over the past years due to the development and approval of different disease-specific drugs. SMA is a rare neuromuscular disorder with the leading symptom of a proximal and progressive muscle weakness. In most cases, SMA is caused by a homozygous deletion in the *survival motor neuron 1* (*SMN1*) gene on chromosome 5 [1]. *SMN2* is a centromeric copy of *SMN1* that produces transcripts of SMN protein lacking exon 7. The result is an alternatively spliced truncated and non-functional SMN protein (SMNΔ7) but to small proportions also functional SMN protein [1, 2]. *SMN2* is expressed in variable copy numbers in patients with SMA, with *SMN2* copy number inversely correlating with disease severity [3]. SMA affects patients of all ages with a broad spectrum of disease severity. In patients with symptom onset later than 6 months of age, a differentiation is made between SMA type 2 (patients gaining the ability to sit unassisted but not to walk) and SMA type 3 (patients gaining the ability to walk unassisted) [4, 5].

For the treatment of SMA patients, three different drugs (nusinersen, onasemnogene abeparvovec, and risdiplam) have been approved with a positive influence on disease progression [6, 7]. While nusinersen was approved in Europe already in 2017, the approval of onasemnogene abeparvovec and risdiplam followed later. Nusinersen is available for the treatment of SMA patients independent of age, *SMN2* copy number, or motor function. As antisense oligonucleotide, nusinersen acts as splicing modifier targeting the intronic splicing silencer N1 in *SMN2* [8]. Sham-controlled, clinical trial data showed that nusinersen treatment significantly improved motor function in children with later-onset SMA aged between 2 and 12 years, with a follow-up period of 15 months [9]. Real-world data from different countries and disease registries could confirm these results in the short-term follow-up [10–14]. Despite data from a small cohort of 15 non-ambulant patients who participated in the phase I/II clinical trials [15], data on the long-term effect of nusinersen in pediatric later-onset SMA patients is still lacking.

The disease-specific SMArtCARE registry aims to collect real-world data on all available SMA patients in Germany, Austria and Switzerland [16]. Here, we report data on pediatric patients with SMA type 2 and 3 with focus on long-term effects on motor, respiratory, and bulbar function.

## Methods

### SMArtCARE registry

With currently 58 participating centers in Germany, Austria and Switzerland, SMArtCARE collects longitudinal data on all available SMA patients as a disease-specific registry. As of November 2021, the registry encompasses data on 1190 patients of any age, SMA type and treatment regime. The only inclusion criteria for patients to be enrolled in SMArtCARE are a genetically confirmed 5q-SMA, and written consent of patients or caregivers. Data are collected during routine patient visits as real-life outcome data. Data are documented using standardized case report forms and not extracted from medical records. Content of these case report forms is aligned with the international consensus for SMA registries [17]. Amongst others, these include information on motor function and motor milestones, respiratory, bulbar and orthopedic symptoms, in addition to adverse events. Genetic test results including *SMN2* copy number are documented by the treating physicians according to the original genetic test results of the patients. *SMN2* copy number is not reassessed centrally within the SMArtCARE registry. Thus, especially *SMN2* copy number is not available for all patients. To evaluate motor function of patients, standardized physiotherapeutic assessments every 4 months are recommended, but are not mandatory within the SMArtCARE data collection und thus not available for all patients at all time-points. Central ethics approval was obtained by the ethics committee of the University of Freiburg (EK-Freiburg 56/18), and local ethics approvals were obtained from all participating centers.

### Patient cohort

In this analysis, we included all non-ambulant patients below 18 years of age with SMA type 2 or type 3, who were treated with nusinersen (data cut 15th of November 2021). Only patients were included with documented baseline characteristics and motor function before start of treatment. Patients were stratified to the following subgroups: “Younger sitters” included all patients with SMA type 2 who were ≤ 5 years and able to sit, but never able to walk independently at start of treatment (n = 107), and “older sitters” included those > 5 years of age (n = 73). “Lost sitters” were all children with SMA type 2 who were never able to walk and lost the ability to sit independently before start of treatment (n = 37), and “lost walkers” were children with SMA type 3 who lost ambulation and partly also the ability to sit independently before start of treatment (n = 39). The first visit of each patient corresponded to treatment initiation with a follow-up of maximum 38 months. The follow-up period of maximum 38 months was chosen, because with the approval of nusinersen in 2017, a conclusive cohort size was still available in all subgroups after 38 months of treatment. According to the different timing of treatment initiation, follow-up times varied and thus not all patients had a follow-up time of 38 months. The last available visit of each patient was considered within the observation period (see Table 1).

**Table 1:** Number of patients per cohort and time-point

	Baseline	m14	m26	m38
Younger sitters	107	87	74	52
Older sitters	73	69	55	40
Lost sitters	37	33	27	15
Lost walkers	39	36	32	22

### Outcomes

Primary outcome of this data analysis were changes in motor function evaluated with the Hammersmith Functional Motor Scale Expanded (HFMSE), the Revised Upper Limb Module (RULM), and motor milestones following WHO criteria [18]. The HFMSE consists of 33 items with a total score of 66 points (higher scores indicating better motor function). A change of ≥ 3 points in the HFMSE score is considered clinically meaningful [19]. The RULM consists of 20 items focusing on changes in upper limb function. The total score is 37 points with a change of ≥ 2 points considered clinically meaningful [20]. Participating physiotherapists were regularly trained to ensure interrater reliability. Further, longitudinal data on the need for ventilator support, the need for tube feeding, and mortality were evaluated. Adverse events (AE) were recorded as AE with or without hospitalization and specified using the Medical Dictionary for Regulatory Activities (MedDRA) code [21]. For each AE, the treating physician was asked to assess whether the AE was related or possibly related to the treatment with nusinersen.

### Statistical analysis

Primary and secondary outcomes and cohorts for subgroup analysis were defined in a statistical analysis plan before data were extracted from the database. Descriptive analysis was performed by calculating absolute frequencies and percentages. Continuous data were analyzed as mean ± standard deviation. Analyses of HFMSE and RULM were additionally based on comparisons of different time-periods: baseline to month 14 (m14), m14 to month 26 (m26), and m26 to month 38 (m38). The last available visit was set as the individual endpoint for each patient and was considered for the analysis of clinically meaningful changes in HFMSE and RULM. For patients who stopped treatment within the 38 months of follow-up, data were considered for analysis maximum 6 months after treatment discontinuation. If patients changed drug treatment, no further data were evaluated after discontinuation of nusinersen treatment. Inferential analyses were applied to evaluate the effect of age at diagnosis, age at start of treatment, *SMN2* copy number, gender, baseline HFMSE or RULM score, and elapsed time from baseline on changes in HFMSE or RULM score. For time-to-event analysis, Kaplan–Meier curves were computed for the probabilities of gaining the ability to walk independently. All curves are presented as cumulative incidence. Statistical analysis was performed using R statistical software (version 4.0.4). A p-value of ≤ 0.01 was considered statistically significant.

## Results

We included data from 256 patients in this analysis. Data were collected and documented at 34 neuropediatric or neurological departments. Table 2 summarizes baseline characteristics of all patients. Younger sitters were considerably younger at start of treatment than children in the other three cohorts. Further, HMFSE and RULM scores at baseline were highest in lost walkers. The need for ventilator support or tube feeding was higher in older and lost sitters, consistent with lower HMFSE scores. In all cohorts, the majority of children had three *SMN2* copies. Before start of treatment, mean age at loss of independent sitting was 29.9 ± 35.7 months in lost sitters and mean age at loss of ambulation was 65 ± 51.1 months in lost walkers. Of all lost walkers, four patients (10.2%) additionally lost the ability to sit independently before start of treatment at a mean age of 63.0 ± 47.9 months.

**Table 2:** Baseline characteristics of all patients

	Younger sitters (n = 107)	Older sitters (n = 73)	Lost sitters (n = 37)	Lost walkers (n = 39)
Age at symptom onset (months)	10.5 ± 5.9	11.3 ± 5.1	10.0 ± 4.4	23.8 ± 16.1
Age at start of treatment (months)	30.8 ± 13.2	120.9 ± 39.1	98.5 ± 61.6	134.7 ± 54.6
*SMN2* copy number				
1 *SMN2*	0 (0%)	1 (1.4%)	0 (0%)	0 (0%)
2 *SMN2*	11 (10.3%)	5 (6.8%)	2 (5.4%)	0 (0%)
3 *SMN*2	78 (72.9%)	37 (50.7%)	23 (62.2%)	26 (66.7%)
≥ 4 *SMN2*	12 (11.2)	11 (15.1%)	3 (8.1%)	6 (15.4%)
Unknown	6 (5.6%)	19 (26.0%)	9 (24.3%)	7 (17.9%)
HFMSE score (n)	20.7 ± 11.4 (52)	15.7 ± 12.4 (47)	13.7 ± 14.4 (15)	25.9 ± 9.1 (33)
RULM score (n)	16.2 ± 7.1 (26)	19.0 ± 7.5 (51)	12.8 ± 7.1 (18)	26.0 ± 6.1 (29)
Sitting without support	100%	100%	0%	89.7%
Non-invasive ventilator support	12 (11.2%)	16 (21.9%)	9 (24.3%)	2 (5.1%)
Tube feeding	4 (3.7%)	5 (6.8%)	5 (13.5%)	0 (0%)
Scoliosis	30 (28.0%)	62 (85.0%)	26 (70.3%)	29 (74.4%)
Contractures	1 (0.9%)	13 (17.8%)	9 (24.3%)	7 (17.9%)

Data are listed as mean ± standard deviation or n (%)

During the observation period, only 13 patients (5.1%) had at least one interval greater than 6 months between nusinersen treatments. Further, of all 2416 documented nusinersen treatments, only 0.8% were given at intervals greater than 6 months. Consequently, despite the impact of COVID-19 pandemia almost all patients received nusinersen treatments at recommended intervals during their follow-up time. Thirteen patients (5.1%) stopped nusinersen treatment. Of these, seven patients (53.5%) changed treatment to risdiplam or onasemnogene abeparvovec. None of the patients received a combination therapy with nusinersen and risdiplam or onasemnogene abeparvovec. Fifteen patients (5.9%) were lost to follow-up with no data entered over more than 12 months.

### Motor milestones

**Fig. 1:**
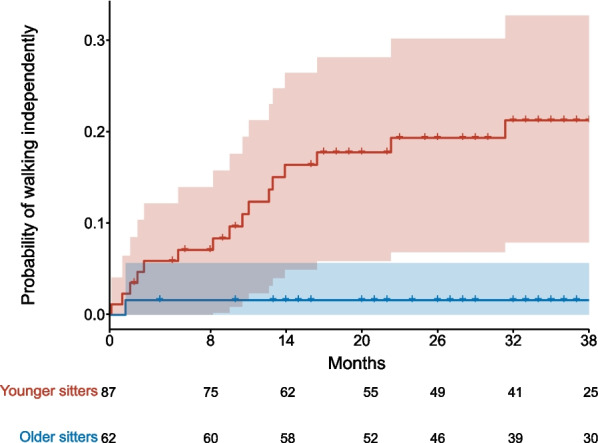
Probability to gain the ability to walk independently. Probability to gain the ability to walk independently under treatment with nusinersen in younger sitters (red) and older sitters (blue). Numbers at risk are listed for dedicated time-points. Colored areas indicate 99% confidence intervals

During the observation period, 16 younger sitters (14.9%) and one older sitter (1.3%) gained the ability to walk independently according to WHO criteria. Of these, 14 children (82.3%) gained the ability to walk between baseline and m14. None of the lost sitters or lost walkers gained the ability to walk unassisted. Figure 1 displays the probability to gain the ability to walk in younger and older sitters. Four lost sitters (10.8%) and one lost walker (2.6%) gained the ability to sit independently. In all cohorts, no motor milestones were lost under nusinersen treatment, in particular no child lost the ability to sit.

### HFMSE

**Fig. 2:**
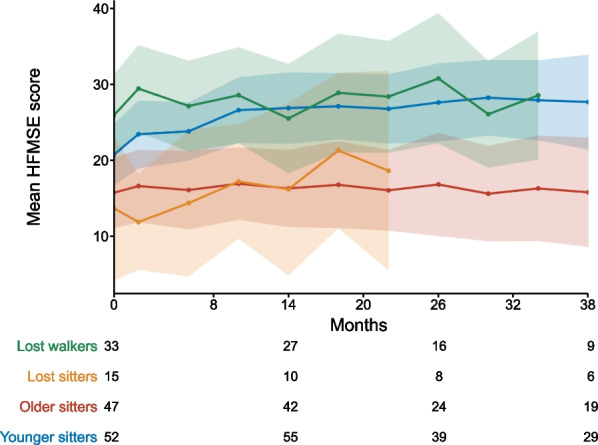
Longitudinal progression of HFMSE score. HFMSE score for younger sitters (blue), older sitters (red), lost sitters (yellow), and lost walkers (green). Data are listed as mean and 99% confidence interval. Available patients at baseline, m14, m26 and m38 are added. For a group size fewer than 10 patients no data are depicted

After 38 months of treatment, changes in HFMSE scores were mean + 7.0 points in younger sitters, + 0.1 points in older sitters and + 2.9 points in lost walkers. The number of lost sitters at m38 was too small to show changes. After 14 months of treatment, mean change in HFMSE was + 2.5 points in lost sitters. Figure 2 illustrates the longitudinal progression of all patients in the different cohorts.

**Fig. 3:**
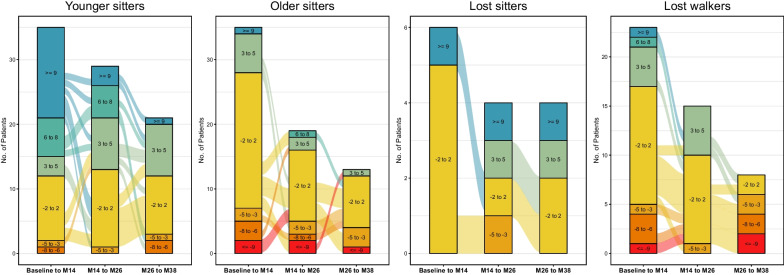
Responder analysis HFMSE. Alluvial diagram to demonstrate changes in HMFSE over time for each cohort. Colors of columns indicate response groups according to changes in HFMSE score per time-period (baseline-m14, m14–m26, m26–m38). Lines between columns indicate the progression between time-periods

Clinically meaningful changes in HFMSE score were observed in 63 children (24.6%): 37 younger sitters (34.6%), 11 older sitters (15.1%), five lost sitters (13.5%), and 10 lost walkers (25.6%). Eleven children (4.3%) lost ≥ 3 points in HFMSE score during the observation period (one younger sitters (1.0%), six older sitters (8.2%) and four lost walkers (10.3%)). Especially in younger sitters, main improvements were observed between baseline and m26: 23 children (21.5%) experienced an improvement ≥ 3 points within the first 14 months, and 16 children (14.9%) between m14 and m26. Only nine children (8.4%) gained ≥ 3 points between m26 and m 38. Figure 3 displays changes in HFMSE score in each cohort.

Inferential analysis revealed *SMN2* copy number as the only covariate having a significant influence on changes in HFMSE score. Further, higher baseline score was associated with smaller improvements in HFMSE score (see Table 3).

**Table 3:** Inferential analysis HFMSE

	Value	SE	DF	t-value	p-value
(Intercept)	1.386	0.697	833	1.987	0.05
Baseline_m14 versus m14_m26	0.659	0.366	833	1.797	0.07
Baseline_m14 versus m26_m38	0.550	0.608	833	0.905	0.37
Age at symptom onset	0.014	0.028	170	0.492	0.62
Baseline score	− 0.044	0.021	170	− 2.119	0.04
***SMN2* copy number**	**1.785**	**0.624**	**170**	**2.861**	**0.005**
Age at start of treatment	− 0.009	0.005	170	− 1.667	0.1
Gender (m)	0.704	0.460	170	1.528	0.13
Loss of sitting before start of treatment	0.170	0.742	170	0.229	0.82
Loss of walking before start of treatment	0.335	0.741	170	0.452	0.65

Inferential analysis evaluates the effect of age at diagnosis, age at start of treatment, *SMN2* copy number (≤ 3 *SMN2* copies vs. ≥ 4 *SMN2* copies), gender, baseline HFMSE score, and past time from baseline on changes in HFMSE score

*SE* Standard error, *DF* degree of freedom

### RULM

**Fig. 4:**
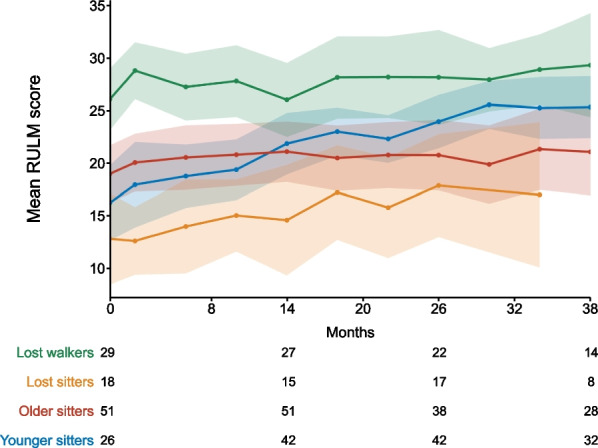
Longitudinal progression of RULM score. RULM score for younger sitters (blue), older sitters (red), lost sitters (yellow) and lost walkers (green). Data are listed as mean and 99% confidence interval. Available patients at baseline, m14, m26 and m38 are added. The difference in cohort size is explained by incomplete data availability for all patients at all time-points. Further, RULM can only be performed in children from an age of 2 years

RULM scores improved in all cohorts. At m38, mean changes in RULM score were + 9.1 points in younger sitters, + 2.2 points in older sitters, + 7.3 points in lost sitters, and + 3.3 points in lost walkers. Figure 4 depicts the difference of the longitudinal progression of patients in the different cohorts.

**Fig. 5:**
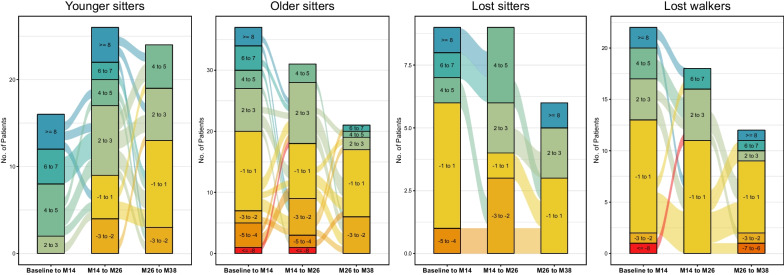
Responder analysis RULM. Alluvial diagram to demonstrate changes in RULM over time for each cohort. Colors of columns indicate response groups according to changes in RULM score per time-period (baseline-m14, m14–m26, m26–m38). Lines between columns indicate the progression between time-periods

Clinically meaningful changes in RULM score were observed in 83 children (32.4%): 30 younger sitters (28.0%), 30 older sitters (41.1%), eight lost sitters (21.6%) and 15 lost walkers (38.5%). Only three older sitters (4.1%) lost ≥ 2 points during the observation period. Improvements in RULM score were observed continuously during the observation period (see Fig. 5).

Inferential analysis revealed the following covariates to have a significant influence on changes in RULM score: Children with higher baseline score showed smaller improvements than children with lower RULM scores. Further, changes in RULM score were observed continuously during the 38 months follow-up (see Table 4).

**Table 4:** Inferential analysis RULM

	Value	SE	DF	t-value	p-value
(Intercept)	3.427	0.776	849	4.416	0.00
**Baseline_m14 versus m14_m26**	**1.122**	**0.235**	**849**	**4.777**	**< 0.001**
**Baseline_m14 versus m26_m38**	**1.509**	**0.364**	**849**	**4.146**	**< 0.001**
Age at symptom onset	0.033	0.027	169	1.235	0.22
**Baseline Score**	**− 0.146**	**0.033**	**169**	− **4.431**	**< 0.001**
*SMN2* copy number	0.465	0.621	169	0.749	0.46
Age at start of treatment	− 0.004	0.005	169	− 0.742	0.46
Gender m	0.396	0.456	169	0.869	0.39
Loss of sitting before start of treatment	− 1.533	0.664	169	− 2.308	0.02
Loss of walking before start of treatment	0.805	0.733	169	1.099	0.27

Inferential analysis evaluates the effect of age at diagnosis, age at start of treatment, *SMN2* copy number (≤ 3 *SMN2* copies vs. ≥ 4 *SMN2* copies), gender, baseline RULM score, and past time from baseline on changes in RULM score.

### Respiratory function and feeding

At baseline, 39 children (15.2%) used part-time (< 16 h per day), non-invasive ventilator support. During the observation period, one older sitter was able to discontinue part-time ventilator support. Twenty-three children (9.0%) additionally started to use occasional ventilator support: two younger sitters (1.9%), 12 older sitters (16.4%), six lost sitters (16.2%) and three lost walkers (7.7%). None of the children required permanent or invasive ventilator support.

At baseline, 14 children (5.5%) required tube feeding due to bulbar dysfunction. During the observation period, five children (2.0%) additionally required tube feeding despite nusinersen treatment: two younger sitters (1.9%), one older sitter (1.4%), and two lost sitters (5.4%). One younger sitter who started tube feeding could discontinue after 4 months.

### Adverse events

In total, 144 AEs among 64 patients were reported during the observation period. Of all AEs, 122 (84.7%) were AEs with hospitalization and 22 (15.3%) without hospitalization. The most common type of AEs were respiratory tract infections (45.8%), followed by gastroenteritis (20.8%), post-lumbar puncture syndrome (9.0%), other type of infections (8.3%), fractures (4.1%), respiratory symptoms including respiratory distress and transient cyanosis (4.2%), pain (3.5%), abdominal symptoms including abdominal pain (1.4%), and others (2.8%). No children died during the observation period. None of the AEs were considered as related to drug treatment, but 31 (25.4%) were rated as possibly related to drug treatment by the treating physician. The latter included procedure-related symptoms as post-lumbar puncture syndrome but also acute infections or respiratory symptoms.

## Discussion

The development and approval of disease modifying treatments have significantly changed the SMA landscape. The longest experience is available for nusinersen treatment, but data are still lacking on long-term benefit and response to treatment for the broad spectrum of SMA patients.

In particular, non-ambulant SMA type 2 and type 3 patients with childhood onset of symptoms have not been extensively studied in long-term, prospectively conducted clinical trials, or in real-world data collections. Phase III clinical trials were limited to patients aged between 2 and 12 years, not having severe contractures or scoliosis, and not using ventilator support more than 6 h per day. In the 84 patients who received nusinersen treatment, motor function improved in the short-term follow-up of 15 months [9]. Data from international disease registries encompass broader patient cohorts with a maximum follow-up period of 24 months and thus still do not provide evidence on the long-term effect of nusinersen treatment [11, 12, 14, 22, 23].

Here, we report real-world data of a broad spectrum of pediatric non-ambulant SMA patients with SMA type 2 or type 3 using a pre-specified analysis. Despite a wide age range, different functional ability at start of treatment reflected by baseline HFMSE and RULM scores in the different cohorts, and different comorbidities (e.g. need for ventilator support or tube feeding), the majority of children experienced a stabilization of disease progression and a great part of children derived clinically meaningful benefit from nusinersen treatment by either gaining a new motor milestone or improving upper limb function or both. In contrast, natural history data of SMA type 2 patients reflect the continuous progression of muscle weakness: within 12 months, a loss of sitting independently in 3.1% of patients and a mean decline in HFMSE score of − 0.54 points within 12 months, and a mean decline in RULM score of − 0.79 points within 24 months were described [24, 25]. Thus, a stabilization of disease status can already be considered as positive response to treatment. We observed main improvements in HFMSE score within the first 24 months of treatment, consistent with data of young SMA type 1 patients [26]. RULM score improved continuously and consistently in all cohorts during the observation period. Especially in older and more severely affected children the performance of HFMSE might be limited due to scoliosis or contractures. Thus, RULM score seems to be preferable for monitoring changes in motor function of these non-ambulant patients.

Despite improvements in motor function, we could not observe a positive effect of nusinersen treatment on the need for non-invasive ventilator support or tube feeding: Our results are in line with results of a previous natural history study, where 38% of sitters required ventilator support with a median age of start of ventilator support of 5.0 years (range 1.8–16.6 years) [27].

Reported AEs from the phase III clinical trial resulted mainly from known side effects of lumbar puncture or were related to the underlying disease with a comparable incidence of AEs in the nusinersen group and the control group. These included amongst others respiratory tract infections, pyrexia, respiratory distress, but also symptoms such as headache, vomiting or back pain [9]. In our pediatric cohort, we did not observe any new safety signals under long-term treatment with nusinersen.

The real-world data approach represents a major advantage, but also a limitation of the present study. Not all data are available for all patients at all time-points. Further, data quality within a registry is not comparable to clinical trials. To ensure high data quality within the SMArtCARE registry, we use standardized case report forms and outcome measures for data collection. Physiotherapists and raters are regularly trained to ensure interrater reliability. In addition, data is carefully reviewed for completeness, consistency and plausibility.

In conclusion, our results demonstrate improvements or stabilization of disease progression in most non-ambulant children with SMA type 2 or type 3 under nusinersen treatment. Changes were most evident in upper limb function while there was no impact on the need for ventilator support or tube feeding.

## Data Availability

All data included in this analysis are recorded in the SMArtCARE registry. Data can be obtained anonymized and aggregated upon request and approval by the SMArtCARE steering committee.
